# Impact of primary tumor sidedness and sex on prognosis and anti-epidermal growth factor receptor antibody efficacy in *BRAF*-mutant metastatic colorectal cancer: a pooled analysis of AIO studies FIRE-1, CIOX, FIRE-3, XELAVIRI, and VOLFI☆

**DOI:** 10.1016/j.esmoop.2024.103677

**Published:** 2024-08-21

**Authors:** A.H.S. Alig, D.P. Modest, S. Stintzing, K. Heinrich, M. Geissler, L. Fischer von Weikersthal, T. Decker, U. Vehling-Kaiser, S. Held, N. Moosmann, A. Stahler, A. Tannapfel, C. Giessen-Jung, A. Jung, L. Weiss, V. Heinemann

**Affiliations:** 1 Charité – Universitätsmedizin Berlin, Corporate Member of Freie Universität Berlin and Humboldt Universität zu Berlin, Department of Hematology, Oncology and Tumorimmunology, Berlin; 2 Department of Medicine III and Comprehensive Cancer Center, University Hospital, LMU Munich, Munich; 3 German Cancer Consortium (DKTK), DKFZ, Heidelberg, Munich; 4 Hospital Karlsruhe, Karlsruhe; 5 Gesundheitszentrum St. Marien, Amberg; 6 Oncological Practice, Ravensburg; 7 ÜBAG MVZ Dr. Vehling-Kaiser GmbH, Landshut; 8 ClinAssess GmbH, Leverkusen; 9 Clinic Barmherzige Brüder Regensburg, Regensburg; 10 Institut für Pathologie der Ruhr-Universität Bochum, Bochum; 11 Institute of Pathology, University of Munich, Munich, Germany

**Keywords:** *BRAF* mutation, EGFR antibody, metastatic colorectal cancer, primary tumor location, primary tumor sidedness

## Abstract

**Background:**

Primary tumor (PT) sidedness is an established prognostic marker in metastatic colorectal cancer (mCRC) and has a predictive impact on the efficacy of anti-epidermal growth factor receptor (anti-EGFR) antibody [monoclonal antibody (mAb)] in patients with *RAS* wild-type mCRC. This investigation focuses on patients with *BRAF*^V600E^-mutated (*BRAF*mt) mCRC and examines the efficacy of anti-EGFR mAbs in relation to primary tumor sidedness (PTS).

**Patient and methods:**

This pooled analysis was carried out using individual patient data from five randomized studies in the first-line setting of mCRC. The population of interest was limited to patients with *BRAF*mt mCRC and known PTS. For analysis, treatment was stratified into two groups: those treated with anti-EGFR mAbs and those without. Dichotomous variables, such as overall response rate and objective response rate (ORR), were compared using chi-square or Fisher’s exact test. Time-to-event endpoints [progression-free survival (PFS) and overall survival (OS)] were analyzed using the Kaplan–Meier method, log-rank test, and Cox regression. An interaction test was carried out via Cox regression.

**Results:**

A total of 102 patients with *BRAF*mt mCRC were identified. The type of targeted therapy (anti-EGFR-based versus non-anti-EGFR) did not significantly impact the outcome. However, in patients with left-sided primary tumors, anti-EGFR mAb-based treatment, compared with non-anti-EGFR, was associated with a higher ORR (58% versus 34%; *P* < 0.01), trended toward improved PFS [hazard ratio (HR) 0.62; 95% confidence interval (CI) 0.34-1.13; *P* = 0.12], and demonstrated prolonged OS (HR 0.38; 95% CI 0.20-0.72; *P* < 0.01). In patients with right-sided primary tumors, anti-EGFR-based therapy had no effect on ORR (33% versus 36%; *P >* 0.99), induced inferior PFS (HR 1.97; 95% CI 1.12-3.47; *P* = 0.02), and trended toward a worse OS (HR 1.76; 95% CI 0.99-3.13; *P* = 0.05).

**Conclusion:**

This analysis suggests that PTS has predictive value for the efficacy of anti-EGFR mAb in the first-line treatment of *BRAF*mt mCRC.

## Highlights

• PTS has no prognostic impact on patients with *BRAF*mt mCRC.
• The prognostic impact of PTS may vary between sexes in *BRAF*mt mCRC.
• Patients with *BRAF*mt mCRC and left-sided primaries may benefit from first-line anti-EGFR-based therapy.

## Background

Initial systemic treatment of metastatic colorectal cancer (mCRC) is based on the tumor’s molecular biology. Upfront testing presently includes not only *RAS* and *BRAF* mutational status, but also analyses of DNA mismatch repair (MMR) or microsatellite instability-high (MSI-H) status. While agents directed against the vascular endothelial growth factor (VEGF) can be used regardless of *RAS* mutation status, agents directed against the epidermal growth factor receptor (EGFR) are not effective in *RAS*-mutated tumors. They are therefore restricted to *RAS* wild-type mCRC.^1^

In mCRC, *BRAF*^V600E^ mutations occur at a rate of 8%-10%.^2^, ^3^, ^4^ *BRAF*^V600E^ and *RAS* mutations are nearly always mutually exclusive.^4^^,^^5^ Patients with proficient MMR and *BRAF*^V600E^-mutated mCRC typically show a poor prognosis and survival times remain in the range of 11-19 months in most studies.^6^, ^7^, ^8^, ^9^

According to a recent meta-analysis of the GONO group, FOLFOX (or FOLFIRI) plus bevacizumab can be regarded as the recommended first-line standard in *BRAF*^V600E^-mutated mCRC, while no increased benefit was observed in this subgroup when the more intensive triplet regimen FOLFOXIRI was applied in combination with bevacizumab.^10^

Although bevacizumab is established in the treatment of *BRAF*^V600E^-mutated mCRC, this is not the case for anti-EGFR monoclonal antibodies (mAbs). In fact, there is an ongoing controversial debate about whether anti-EGFR agents are not only ineffective but may even be harmful in this subgroup.^11^^,^^12^ In the recently published FIRE-4.5 study, FOLFOXIRI combined with anti-EGFR mAb was inferior to FOLFOXIRI in combination with bevacizumab.^13^

A more differentiated analysis of a potentially complex situation was made possible by a subgroup evaluation of FIRE-3, where patients with *BRAF*^V600E-^mutated mCRC were evaluated according to response dynamics.^9^ Early tumor shrinkage (ETS) was achieved in 53% in the cetuximab arm and 33% in the bevacizumab arm. ETS compared with no ETS was associated with a favorable outcome [overall survival (OS) 29.8 versus 5.9 months] in cetuximab-treated, but not in bevacizumab-treated patients (11.8 months versus 13.7 months).^9^ This analysis demonstrates that *BRAF*^V600E^-mutated mCRC is a heterogeneous disease with distinctly different patterns of response to anti-EGFR and anti-VEGF agents. The heterogeneity of *BRAF*^V600E^-mutated mCRC has been well established by Barras et al.^3^ and Guinney et al.^14^ describing distinct molecular subgroups within the population of patients with *BRAF*^V600E^-mutated mCRC according to their gene expression profile. Those subtypes differ in their prognosis and potentially in the efficacy of treatments.

The present analysis targets the biological heterogeneity of *BRAF*^V600E^-mutated mCRC with a specific focus on primary tumor sidedness (PTS). To increase the strength of the analysis, individual patient data from five randomized first-line studies were included.

## Methods

### Trials

The present analysis includes individual patient data from five randomized prospective AIO trials (FIRE-1, CIOX, FIRE-3, XELAVIRI, and VOLFI) carried out in the first-line treatment setting of mCRC. All trials were conducted according to the Declaration of Helsinki and were approved by ethics committees. Detailed reports of all trials have been published previously.^9^^,^^15^, ^16^, ^17^, ^18^, ^19^, ^20^, ^21^, ^22^, ^23^, ^24^, ^25^ Supplementary Table S1, available at https://doi.org/10.1016/j.esmoop.2024.103677, provides an overview of the included studies.

### Patients

A pseudonymized clinical database of the trials with the preselection of *BRAF*^*V600E*^-mutant/*RAS* wild-type disease was established including the following information for each patient: trial, treatment arm, use of EGFR antibody, age, sex, Eastern Cooperative Oncology Group (ECOG) performance status, tumor characteristics (primary tumor sidedness and metastatic sites), and prior adjuvant treatment. Tumor samples assigned to each patient were tested for mutational status (*RAS* and *BRAF*) as described previously.^9^^,^^17^, ^18^, ^19^, ^20^, ^21^, ^22^, ^23^^,^^25^^,^^26^

### Primary tumor sidedness and location

Information on primary tumor sidedness was extracted from the respective study report forms. Primary tumors were classified as right- versus left-sided mCRC with a cut-off at the splenic flexure. Patients with more than one primary tumor were excluded from the analysis.

### Treatment

Treatment procedures were described in previous publications and are summarized in Supplementary Table S1, available at https://doi.org/10.1016/j.esmoop.2024.103677.^9^^,^^17^, ^18^, ^19^, ^20^, ^21^, ^22^, ^23^^,^^25^^,^^26^ For further analysis, treatment was stratified into two groups: treatment with or without anti-EGFR antibody. The latter group comprises chemotherapy regimens with or without the VEGF inhibitor bevacizumab.^9^^,^^17^, ^18^, ^19^, ^20^, ^21^, ^22^, ^23^^,^^25^^,^^26^

### Definition of efficacy endpoints

Objective response rate (ORR) was evaluated according to the World Health Organization (WHO) classifications (FIRE-1), RECIST 1.0 (CIOX, FIRE-3), or RECIST 1.1 (XELAVIRI, VOLFI). Progression-free survival (PFS) was defined as the time from randomization to the first progression of disease or death from any cause. In addition, the XELAVIRI study defined PFS as time from randomization to failure of strategy, meaning switching to an anticancer drug not included in the XELAVIRI study. OS was defined as time from randomization until death from any cause.

### Statistical analysis

All statistical analyses were carried out using SAS software (version 9.4; SAS Institute, Cary, NC) and SPSS version 28.0 software (IBM Corporation, Armonk, NY). Survival was expressed as medians including 95% confidence intervals (CIs) by the Kaplan–Meier method and compared using log-rank tests. In addition, Cox regression analyses with maximum-likelihood estimation were used for interaction testing. Dichotomous variables were compared by Fisher’s exact test or the chi-square test. Odds ratios were indicated when appropriate with 95% CIs. The two-sided significance level was set to 0.05 and estimates are reported with 95% CI.

## Results

### Population

Out of the five trials, 1393 patients with known mutational status were identified. A total of 102 patients (7.3%) had *BRAF*^V600E^-mutant/*RAS* wild-type (*BRAF*mt) tumors with known primary tumor location. Patients with more than one primary tumor were excluded from the analysis. Please also refer to Supplementary Figure S1, available at https://doi.org/10.1016/j.esmoop.2024.103677.

### Patient and tumor characteristics

The baseline characteristics of the 102 patients with *BRAF*mt mCRC are summarized in Table 1. In this population, 55 patients (54%) presented with right-sided primary tumors (RSPTs) and 47 (46%) with left-sided primary tumors (LSPTs). PTS was statistically significantly associated with sex. Patients with RSPT were more likely to be female (*P* = 0.04).

**Table 1:** Patient and tumor characteristics of *BRAF*^V600E^ mutant/*RAS* wild-type population according to primary tumor sidedness

Characteristics	*BRAF*mt population (*n* = 102), *n* (%)	Left-sided primaries (*n* = 47), *n* (%)	Right-sided primaries (*n* = 55), *n* (%)
**Study**			
FIRE-1	5 (5)	3 (6)	2 (4)
CIOX	17 (17)	6 (13)	11 (20)
FIRE-3	47 (46)	23 (49)	24 (44)
XELAVIRI	19 (19)	9 (19)	10 (18)
VOLFI	14 (14)	6 (13)	8 (15)
**Sex**			
Male	56 (55)	31 (66)	25 (45)
Female	46 (45)	16 (34)	30 (55)
**Age (years)**			
≤70	77 (75)	38 (81)	39 (71)
>70	25 (25)	9 (19)	16 (29)
**ECOG**			
0	53 (52)	21 (45)	32 (58)
1	47 (46)	25 (53)	22 (40)
2	2 (2)	1 (2)	1 (2)
**Antibody**			
None	13 (13)	7 (15)	6 (11)
Anti-EGFR	46 (45)	21 (45)	25 (45)
Anti-VEGF	43 (42)	19 (40)	24 (44)
**Metastatic spread**			
Liver	72 (71)	30 (64)	42 (76)
Liver-limited	25 (25)	11 (23)	14 (25)
Lung	29 (28)	15 (32)	14 (25)
Lymph nodes	51 (50)	21 (45)	30 (55)
Peritoneum	19 (19)	9 (19)	10 (18)
**Number of metastatic sites**			
1	36 (35)	19 (40)	17 (27)
≥2	56 (55)	23 (49)	33 (60)
Unknown	10 (10)	5 (11)	5 (9)
**Onset of metastases**			
Synchronous	55 (54)	23 (49)	32 (58)
Metachronous	15 (15)	12 (26)	3 (5)
Unknown	32 (31)	12 (26)	20 (36)
**Previous chemotherapy**			
No	86 (84)	38 (81)	48 (87)
Yes	15 (15)	9 (19)	6 (11)
Unknown	1 (1)	0 (0)	1 (2)

*BRAF*mt, *BRAF*^V600E^ mutant/*RAS* wild-type.

### Prognostic impact of primary tumor sidedness

**Figure 1:**
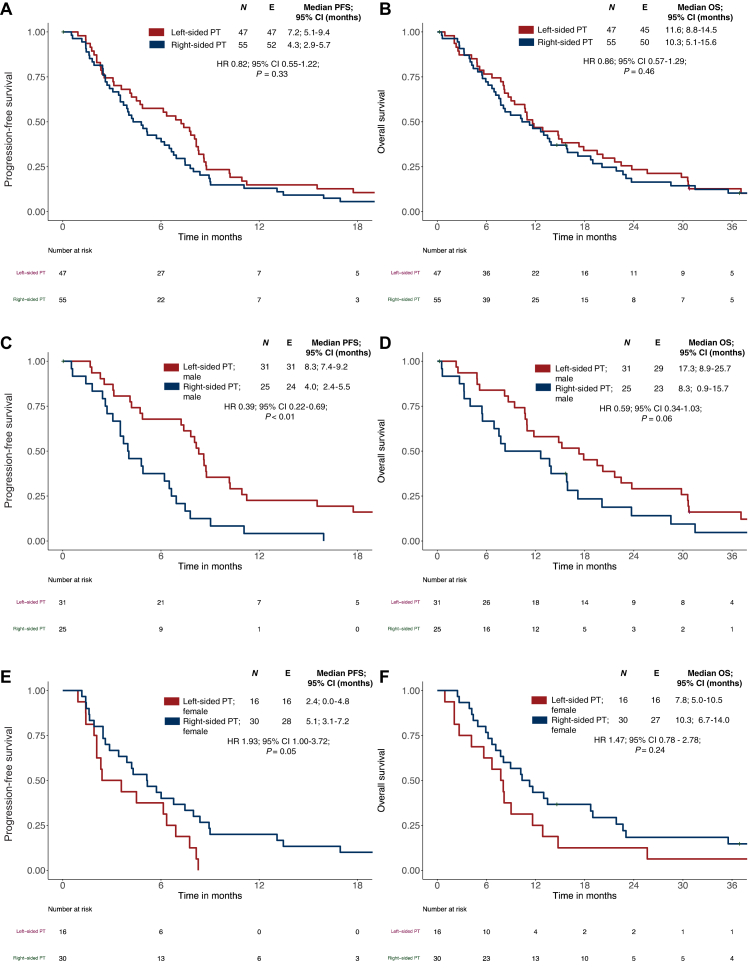
**Progression-free survival (PFS) and overall survival (OS) according to primary tumor sidedness and sex in *BRAF*mt metastatic colorectal cancer (mCRC)**. (A) PFS according to primary tumor sidedness in *BRAF*mt mCRC. (B) OS according to primary tumor sidedness in *BRAF*mt mCRC. (C) PFS in male patients with *BRAF*mt mCRC according to primary tumor sidedness. (D) OS in male patients with *BRAF*mt mCRC primary tumor sidedness. (E) PFS in female patients with *BRAF*mt mCRC according to primary tumor sidedness. (F) OS in female patients with *BRAF*mt mCRC according to primary tumor sidedness. *P* values correspond to Cox regression. *BRAF*mt, *BRAF*^V600E^ mutant/*RAS* wild-type; CI, confidence interval; HR, hazard ratio; OS, overall survival; PT, primary tumor.

In the overall population of *BRAF*mt mCRC, ORR was superior in patients with LSPT compared with RSPT without being statistically significant (LSPT: 55% versus RSPT: 35%; *P* = 0.05). Numerically more favorable data were obtained in LSPT with regard to PFS (LSPT: 7.2 months versus RSPT: 4.3 months; hazard ratio (HR) 0.82; 95% CI 0.55-1.22; *P* = 0.33) and OS (LSPT: 11.6 months versus RSPT: 10.3 months; HR 0.86; 95% CI 0.57-1.29; *P* = 0.46; see Figure 1A and B).

The multivariate analysis for OS in LSPT revealed a significant association with sex (*P* = 0.01); however, in RSPT no significant association was found. To better understand the impact on outcome according to PTS and sex, we analyzed the outcome in LSPT and RSPT according to sex:

In male patients, LSPT compared with RSPT was associated with superior outcome parameters: there was a trend regarding ORR (LSPT: 71% versus RSPT: 45%; *P* = 0.09), a statistically relevant improvement of PFS (HR 0.39; 95% CI 0.22-0.69; *P* < 0.01), and clinically meaningful trend in OS (HR 0.59; 95% CI 0.34-1.03; *P* = 0.06).

A reverse pattern was observed in female patients. ORR was comparable, regardless of sidedness (LSPT: 25% versus RSPT: 27%, *P >* 0.99), but PFS (HR 1.93; 95% CI 1.00-3.72; *P* = 0.05) and OS rather favored RSPT without being statistically relevant (HR 1.47; 95% CI 0.78-2.78; *P* = 0.24). The Kaplan–Meier curves are indicated in Figure 1C–F.

Data obtained in *BRAF*mt mCRC suggest that LSPT is associated with better outcomes in male, but not in female patients.

### Predictive value of sidedness of primary tumor

Of 102 patients with *BRAF*mt mCRC, 45% (*n* = 46) received anti-EGFR-based first-line therapy, while 55% (*n* = 56) did not. In Supplementary Table S2, available at https://doi.org/10.1016/j.esmoop.2024.103677, baseline patient and tumor characteristics of the treatment groups are shown.

**Figure 2:**
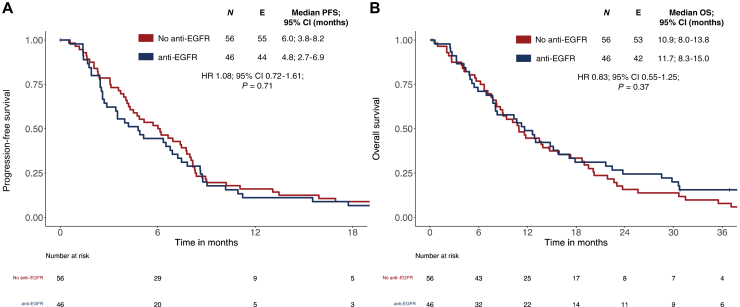
**Progression-free survival (PFS) and overall survival (OS) in *BRAF*mt metastatic colorectal cancer (mCRC) cases when treated with or without anti-EGFR.** (A) PFS according to treatment with or without anti-EGFR in *BRAF*mt mCRC. (B) OS according to treatment with or without anti-EGFR in *BRAF*mt mCRC. *P* values correspond to Cox regression. *BRAF*mt, *BRAF*^V600E^ mutant/*RAS* wild-type; CI, confidence interval; EGFR, epidermal growth factor receptor; HR, hazard ratio.

ORR in the *BRAF*mt patients was higher in patients treated with anti-EGFR-based therapy (58% versus 34%; *P* = 0.02). Analysis of the overall population showed comparable PFS and OS independent of having received anti-EGFR agents or not (see Figure 2A and B).

In LSPT patients with *BRAF*mt tumors, anti-EGFR mAb-based treatment versus none was associated with higher ORR (81% versus 35%; *P* < 0.01), more favorable OS (HR 0.38; 95% CI 0.20-0.72; *P* < 0.01) and a trend toward improved PFS (HR 0.62; 95% CI 0.34-1.13; *P* = 0.12).

**Figure 3:**
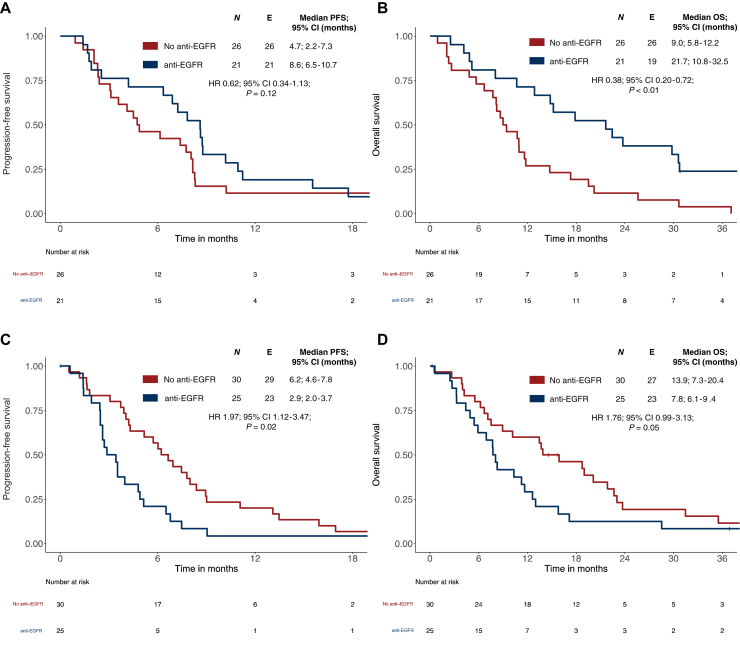
**Progression-free survival (PFS) and overall survival (OS) in *BRAF*mt metastatic colorectal cancer (mCRC) cases according to primary tumor sidedness and treatment with or without anti-EGFR.** (A) PFS in left-sided primary tumor (LSPT) according to treatment with or without anti-EGFR in *BRAF*mt mCRC. (B) OS in LSPT according to treatment with or without anti-EGFR in *BRAF*mt mCRC. (C) PFS in right-sided primary tumor (RSPT) according to treatment with or without anti-EGFR in *BRAF*mt mCRC. (D) OS in RSPT according to treatment with or without anti-EGFR in *BRAF*mt mCRC. *P* values correspond to Cox regression. *BRAF*mt, *BRAF*^V600E^ mutant/*RAS* wild-type; CI, confidence interval; EGFR, epidermal growth factor receptor; HR, hazard ratio.

By contrast, this effect was not observed in patients with RSPT with *BRAF*mt tumors. In this subgroup, anti-EGFR-based therapy had no positive impact on ORR (36% versus 33%; *P >* 0.99), induced inferior results with regard to PFS (HR 1.97; 95% CI 1.12-3.47; *P* = 0.02), and trended toward worse OS (HR 1.76; 95% CI 0.99-3.13; *P* = 0.05). The respective Kaplan–Meier curves are indicated in Figure 3A–D.

With regard to OS, the interaction of sidedness and anti-EGFR antibody efficacy was also present when the analysis was restricted to patients with *BRAF*^V600E^-mutated mCRC from FIRE-3 and VOLFI. These studies represent the purest population as they contained direct randomization of anti-EGFR-based treatment versus no antibody or bevacizumab (*P* < 0.01). The Kaplan–Meier curves of the FIRE-3/VOLFI subset are shown in Supplementary Figure S2A and B, available at https://doi.org/10.1016/j.esmoop.2024.103677.

An interaction test revealed significant results for OS and showed a trend toward significance for PFS between primary tumor sidedness and the treatment arms in the respective study (OS: *P* = 0.01; PFS: *P* < 0.01); additionally, a significant interaction was observed between anti-EGFR and non-anti-EGFR treatment and PTS for both OS (*P* < 0.010) and PFS (*P* = 0.05). The interaction test for OS was significant between anti-EGFR and non-anti-EGFR treatments and PTS when the analysis was restricted to patients from the VOLFI and FIRE-3 studies (*n* = 61, *P* = 0.03).

## Discussion

The presented analysis, based on five randomized trials including data from 102 patients with *BRAF*mt mCRC out of 1393 patients with known molecular subtype, represents an adequate basis for evaluating the prognostic and predictive effects of the primary tumor’s sidedness in *BRAF*mt mCRC. However, it is important to clarify that the data come from five pooled trials, each using different therapeutic strategies with various chemotherapy regimens and antibody usage. Currently, some of these regimens are no longer considered standard of care for this population.

In our analysis, patients with *BRAF*mt mCRC with LSPTs and RSPTs showed no difference in PFS and OS overall.

Interestingly, primary tumor sidedness predicted the efficacy of anti-EGFR antibody therapy in our *BRAF*mt mCRC cohort in terms of ORR and OS. LSPTs appeared to benefit from anti-EGFR therapy, whereas RSPTs did not. However, anti-EGFR antibodies did not affect PFS in our cohort. Whether PFS is a good marker for defining the efficacy of anti-EGFR mAb has been questioned in previous publications,^27^^,^^28^ and it has been suggested that anti-EGFR antibodies may instead impact ORR and OS.^19^^,^^23^^,^^29^, ^30^, ^31^ Similar combinations with increased ORR and prolonged OS, even in the absence of clinically relevant effects on PFS, have been frequently observed in an evidently anti-EGFR-sensitive population (i.e. *RAS/BRAF* wild-type mCRC).^23^^,^^32^, ^33^, ^34^ The stronger beneficial effect on ORR compared with OS for anti-EGFR antibody treatment could stem from rapidly developing resistance to anti-EGFR medication in *BRAF*mt mCRC. Therefore it could be hypothesized that the presence of *BRAF*^V600E^ mutations may not necessarily predict a lack of anti-EGFR antibody efficacy. Our findings suggest that patients with *BRAF*mt mCRC originating from LSPTs may benefit from anti-EGFR antibodies to a similar extent as those with *RAS/BRAF* wild-type tumors. This raises the question of whether there are patients with left-sided *BRAF*mt mCRC who could benefit from anti-EGFR-containing first-line therapy, especially when combined with *BRAF* inhibition.

However, it should be noted that this pooled analysis is limited by its retrospective and exploratory design, as well as by the low number of patients, which restricts the ability to draw definite conclusions. Further, there may be confounding aspects influencing these results. In particular, the missing information on mutational status in the analyzed studies (27%, see Supplementary Figure S1, available at https://doi.org/10.1016/j.esmoop.2024.103677) and the overall low prevalence of *BRAF* mutations in mCRC may introduce bias.

The metastatic spread of right-sided tumors compared with left-sided ones may also introduce bias. In our analysis, left-sided tumors had a higher likelihood of having only one metastatic side and of involving pulmonary metastases, thus indicating a lower overall tumor load. This could disadvantage patients with RSPTs. In addition, patients with LSPTs were younger, potentially leading to a healthier patient population and a higher percentage of second-line therapies, which might account for the discordance in PFS and OS results. However, this does not explain the higher ORR observed with anti-EGFR treatment.

However, patients treated with anti-EGFR-containing regimens were from more recent studies, whereas 27% of the study population treated with non-anti-EGFR regimens were part of the FIRE-1 (mIROX) and XELAVIRI (sequential treatment option, see Supplementary Table S1 and S2, available at https://doi.org/10.1016/j.esmoop.2024.103677). These therapeutic options are now considered suboptimal and may also contribute to bias.

Thus the presented results might be secondary to the generally better prognosis of left-sided tumors, which could be more pronounced in the optimally treated patient population. Nevertheless, the interaction test for OS between primary tumor sidedness and the applied treatment (including respective treatment arms and anti-EGFR versus non-anti-EGFR containing therapy) was positive.

The applied second-line therapies could introduce bias, potentially distorting OS.

Furthermore, pooling data from five studies, two of which directly randomized anti-EGFR antibodies and each using different treatment regimens, may have introduced potential undetected biases due to the heterogeneity of the populations.

Our findings contrast with reports identifying classical *BRAF*^V600E^ mutations as likely negative predictors of anti-EGFR-directed therapy in chemotherapy-treated cohorts.^11^^,^^35^^,^^36^ However, it could be argued that these studies did not adjust their analyses for sidedness and might therefore be biased by the relative over-representation of *BRAF*^V600E^ mutations in right-sided colon segments.^4^

Although the FIRE-4.5 trial demonstrated a detrimental effect of anti-EGFR antibodies when combined with FOLFOXIRI, RSPTs appear to gain benefit more from FOLFOXIRI combined with bevacizumab regarding ORR, PFS, and OS. However, this effect was not observed in LSPTs.^13^ In the FIRE-4.5 trial, the triplet chemotherapy FOLFOXIRI was used as the backbone, whereas this analysis primarily includes patients treated with doublet chemotherapy. Thus any additional lack of benefit derived from adding anti-EGFR in LSPTs could be partially linked to the chemotherapy backbone, similar to *RAS*/*BRAF* wild-type tumors.^37^ Even if this hypothesis is valid, this analysis does not address whether adding anti-EGFR in the first-line setting would impact the efficacy of the approved second-line treatment with encorafenib and cetuximab. Consecutively, current guideline recommendations^38^ do not recommend anti-EGFR-targeted antibody treatment as part of first-line therapy, with cetuximab currently being used primarily in combination with a *BRAF* inhibitor for refractory patients.^39^

Moreover, if the BREAKWATER trial, which examines the role of combining anti-*BRAF* and anti-EGFR treatments with chemotherapy in the first-line setting, yields positive results, then this analysis may become less relevant.

As already mentioned, there is a vast clinical heterogeneity among patients with *BRAF*mt mCRC.^3^^,^^14^ Therefore to better predict the individual disease courses, clinical scoring systems with prognostic significance have been developed.^40^ Several molecular factors contributing to this heterogeneity have been described: a higher plasmatic *BRAF*^V600E^ allele frequency is associated with more aggressive disease and worse outcomes, likely due to the associated higher tumor load.^41^ In addition, some distinct genomic alterations, such as RNF43 mutations, have been identified as having prognostic and predictive impacts with regard to the efficacy of systemic and/or targeted therapies.^42^ Furthermore, MSI status is a crucial prognostic and predictive marker in the era of immunotherapy for patients with mCRC. Patients with *BRAF*mt MSI-H mCRC have significantly worse outcomes when treated with chemotherapy compared with immunotherapy.^43^^,^^44^ MSI-H tumors occur in ∼21% of *BRAF*mt mCRC cases and are more frequently found in right-sided tumors, which could be a relevant negative confounder in this chemotherapy-treated cohort, especially because MSI status is missing.^45^ In addition, different molecular subgroups could explain the varied prognostic impact of primary tumor sidedness between sexes, which could remain undetected in this analysis.

Nevertheless, primary tumor sidedness is a known prognostic marker in *RAS/BRAF* wild-type mCRC, serving as a surrogate of underlying molecular alterations. The ease of determining primary tumor sidedness is an advantage and could have clinical significance in *BRAF*mt mCRC.

The results of our analysis should be interpreted as hypothesis-generating and need confirmation from further trials with direct anti-EGFR antibody randomization, such as CRYSTAL, PRIME, OPUS, CALGB/SWOG 80405, and PEAK.^46^, ^47^, ^48^, ^49^ These trials should pay special attention to microsatellite status, prognostic groups, and sex.

### Conclusions

In our analysis, patients with *BRAF*^V600E^-mutant, *RAS* wild-type mCRC with left-sided primaries had a numerically better prognosis, and a beneficial effect from first-line anti-EGFR-based therapy is suspected. Moreover, the prognostic impact of primary tumor sidedness may differ between sexes.
