# Supplementary material for: Impact of primary tumor sidedness and sex on prognosis and anti-epidermal growth factor receptor antibody efficacy in BRAF-mutant metastatic colorectal cancer: a pooled analysis of AIO studies FIRE-1, CIOX, FIRE-3, XELAVIRI, and VOLFI
Source: ESMO Open. 2024 Aug 21;9(9):103677. doi: 10.1016/j.esmoop.2024.103677 (PMC11387224; doi:10.1016/j.esmoop.2024.103677)
Supplement: Supplementary data [file mmc1.docx]

**Supplementary Material**

**Supplementary Table 1: study characteristics**

|  | **FIRE-1** | **CIOX** | **FIRE-3** | **XELAVIRI** | **VOLFI** |
| --- | --- | --- | --- | --- | --- |
| **pts. included in pooled analysis, n** | 5 | 17 | 47 | 19 | 14 |
| **Phase of study** | III | II | III | III | II |
| **Recruiting period and country** | 07/2000 -10/2004  Germany | 09/2004 -12/2006  Germany | 01/2007 - 09/2012  Germany / Austria | 12/2010 - 04/2016  Germany | 06/2011 - 01/2016  Germany |
| **Primary endpoint** | PFS | ORR | ORR | TFS | ORR |
| **OS censored*** | 12.9 % | 19.8 % | 12.7 % | 15.2 % | 34.4 % |
| **Treatment arms** |  |  |  |  |  |
| Arm A | FUFIRI | CAPIRI+Cet | FOLFIRI+Cet | FP+Bev -> PD  -> FP+Iri+Bev | mFOLFOXIRI+  Pani |
| Arm B | mIROX | CAPOX+Cet | FOLFIRI+Bev | FP+Iri+Bev | FOLFOXIRI |
| **Molecular subgroup allowed** | all | all | All; KRAS exon 2 WT from 2009 | all | KRAS exon 2 WT; RAS WT from 2014 |
| **Previous adj. ctx. allowed**** | yes (no TOP1 inhib., no platinum) | yes (no TOP1 inhib.) | yes | yes | yes |
| **RECIST version** | - (WHO) | 1.0 | 1.0 | 1.1 | 1.1 |
| **Trial registration** | - | NCT00254137 | NCT00433927 | NCT01249638 | NCT01328171 |
| **Eligibility criteria** |  |  |  |  |  |
| Age, years | 18-75 | 18-75 | 18-75 | ≥ 18 | ≥ 18 |
| ECOG | - | - | ≤ 2 | ≤ 1 | ≤ 1 |
| Karnowsky | ≥ 70% | ≥ 70% | - | - | - |

**Legend:** Adj.=adjuvant; Bev=bevacizumab; CAPIRI=oral fluorouracil and irinotecan; CAPOX=oral fluorouracil and oxaliplatin; Cet=cetuximab; ctx.=chemotherapy; FOLFIRI=infusional fluorouracil, leucovorin, and irinotecan; FOLFOX=infusional fluorouracil, leucovorin, and oxaliplatin; FOLFOXIRI=infusional fluorouracil, leucovorin, oxaliplatin and irinotecan; FUFIRI=irinotecan, leucovorin and infusional fluoruracil; inhib.=inhibitors; Iri=irinotecan, FP=fluorouracil (oral or infusional, infusional with leucovorin); mFOLFOXIRI=modified FOLFOXIRI; mIROX=irinotecan and oxaliplatin; min.=minimal; n=number; ORR=objective response rate; OS=overall survival; PD=progressive disease; PFS=progression free survival; Pts=patients; TFS=time to failure of strategy, TOP1=topoisomerase 1; WT=wild-type.

**in final data base; **interval between end of study and recurrence had to be at least 6 months in every study*

**Supplementary Table 2: Patient and tumor characteristics of BRAF^V600E^ mutant/RAS wild-type population according to anti-EGFR or no anti-EGFR containing therapy**

| **Characteristics** | **No-anti-EGFR**  **(n=56)** | | **Anti-EGFR**  **(n=46)** | | **BRAFmt population**  **(n=102)** | |
| --- | --- | --- | --- | --- | --- | --- |
| **Treatment (study)** | | | | | | |
| ***FIRE-1*** |  |  |  |  |  |  |
| FUFIRI | 0 | (0%) | 0 | (0%) | 0 | (0%) |
| mIROX | 5 | (9%) | 0 | (0%) | 5 | (5%) |
| ***CIOX*** |  |  |  |  |  |  |
| CAPIRI+Cet | 0 | (0%) | 9 | (20%) | 9 | (9%) |
| CAPOX+Cet | 0 | (0%) | 8 | (17%) | 8 | (8%) |
| ***FIRE-3*** |  |  |  |  |  |  |
| FOLFIRI+Cet | 24 | (43%) | 0 | (0%) | 24 | (24%) |
| FOLFIRI+Bev | 0 | (0%) | 23 | (50%) | 23 | (23%) |
| ***XELAVIRI*** |  |  |  |  |  |  |
| Sequential arm* | 10 | (18%) | 0 | (0%) | 10 | (10%) |
| Combination arm** | 9 | (16%) | 0 | (0%) | 9 | (9%) |
| ***VOLFI*** |  |  |  |  |  |  |
| mFOLFOXIRI+Pani | 0 | (0%) | 6 | (13%) | 6 | (6%) |
| FOLFOXIRI | 8 | (14%) | 0 | (0%) | 8 | (8%) |
| **Sex** | | | | | | |
| Male | 25 | (45%) | 31 | (67%) | 56 | (55%) |
| Female | 31 | (55%) | 15 | (33%) | 46 | (45%) |
| **Age (years)** | | | | | | |
| ≤ 70 | 40 | (71%) | 37 | (80%) | 77 | (75%) |
| >70 | 16 | (29%) | 9 | (20%) | 25 | (25%) |
| **ECOG** | | | | | | |
| 0 | 32 | (57%) | 21 | (46%) | 53 | (52%) |
| ≥1 | 24 | (43%) | 25 | (54%) | 49 | (48%) |
| **Primary tumour side** | | | | | | |
| Left-sided | 26 | (46%) | 21 | (46%) | 47 | (46%) |
| Right-sided | 30 | (53%) | 25 | (54%) | 55 | (54%) |
| **Metastatic spread** | | | | | | |
| Liver | 34 | (61%) | 38 | (83%) | 72 | (71%) |
| Liver-limited | 14 | (25%) | 11 | (24%) | 25 | (25%) |
| Lung | 14 | (25%) | 15 | (33%) | 29 | (28%) |
| Lymph nodes | 26 | (46%) | 25 | (54%) | 51 | (50%) |
| Peritoneum | 10 | (18%) | 9 | (20%) | 19 | (19%) |
| **No. of metastatic sites** | | | | | | |
| 1 | 23 | (41%) | 13 | (28%) | 36 | (33%) |
| ≥ 2 | 29 | (52%) | 27 | (67%) | 56 | (55%) |
| Unknown | 4 | (7%) | 6 | (4%) | 10 | (10%) |
| **Onset of metastases** | | | | | | |
| Synchronous | 37 | (66%) | 18 | (39%) | 55 | (54%) |
| Metachronous | 10 | (18%) | 5 | (11%) | 15 | (15%) |
| Unknown | 9 | (16%) | 23 | (50%) | 32 | (31%) |
| **Previous chemotherapy** | | | | | | |
| No | 46 | (82%) | 40 | (87%) | 86 | (84%) |
| Yes | 9 | (16%) | 6 | (13%) | 15 | (15%) |
| Unknown | 1 | (2%) | 0 | (0%) | 1 | (1%) |

**Legend:** CAPIRI=oral fluorouracil and irinotecan; CAPOX=oral fluorouracil and oxaliplatin; Cet=cetuximab; FOLFIRI=infusional fluorouracil, leucovorin, and irinotecan; FOLFOX=infusional fluorouracil, leucovorin, and oxaliplatin; FOLFOXIRI=infusional fluorouracil, leucovorin, oxaliplatin and irinotecan; FUFIRI=irinotecan, leucovorin and infusional fluorouracil; mFOLFOXIRI=modified FOLFOXIRI; mIROX=irinotecan and oxaliplatin; no., number.

*Sequential arm*: fluoropyrimidines (oral or infusional, infusional with leucovorin) in combination with bevacizumab until progression, then adding irinotecan.*

*Combination arm**: upfront combination treatment with fluoropyrimidines, irinotecan and bevacizumab (if at least stable disease was achieved after 6 months de- and re-escalation of irinotecan was allowed*

**Supplementary Figure 1: consort diagram**

Patients of FIRE-1, CIOX, FIRE-3, XELAVIRI, VOLFI

**(n=1.908)**

Patients with known BRAFmt status

**(n=109)**

Patients with known primary tumor location, without RAS and with BRAF mutation

**(n=102)**

Exclusion of patients with unknown mutational status (n=515)

Exclusion of patients without known primary tumor site and/or >1 primary tumor

(n=7)

Patients with known mutational status

**(n=1.393)**

Exclusion of patients with mutations other than BRAFV600E (n=1.284)

**Supplementary Figure 2: OS according to treatment with or without anti-EGFR mAb regarding sidedness in the subset of patients treated in the FIRE-3 and VOLFI study**

**A** - OS in LSPT according to treatment with or without anti-EGFR in BRAFmt mCRC

**B** - OS in RSPT according to treatment with or without anti-EGFR in BRAFmt mCRC

**Legend:** 95% CI, 95% confidence interval; *BRAF*mt, *BRAF*^V600E^ mutant/*RAS* wild-type; HR, hazard ratio; LPST, left-sided primary tumor; mCRC, metastatic colorectal cancer; OS, overall survival; RSPT, right-sided primary tumor. p-values: cox regression.
