# Supplementary material for: Impact of primary tumor sidedness and sex on prognosis and anti-epidermal growth factor receptor antibody efficacy in BRAF-mutant metastatic colorectal cancer: a pooled analysis of AIO studies FIRE-1, CIOX, FIRE-3, XELAVIRI, and VOLFI
Source: ESMO Open. 2024 Aug 21;9(9):103677. doi: 10.1016/j.esmoop.2024.103677 (PMC11387224; doi:10.1016/j.esmoop.2024.103677)
Supplement: Supplementary Figures [file mmc2.pdf]

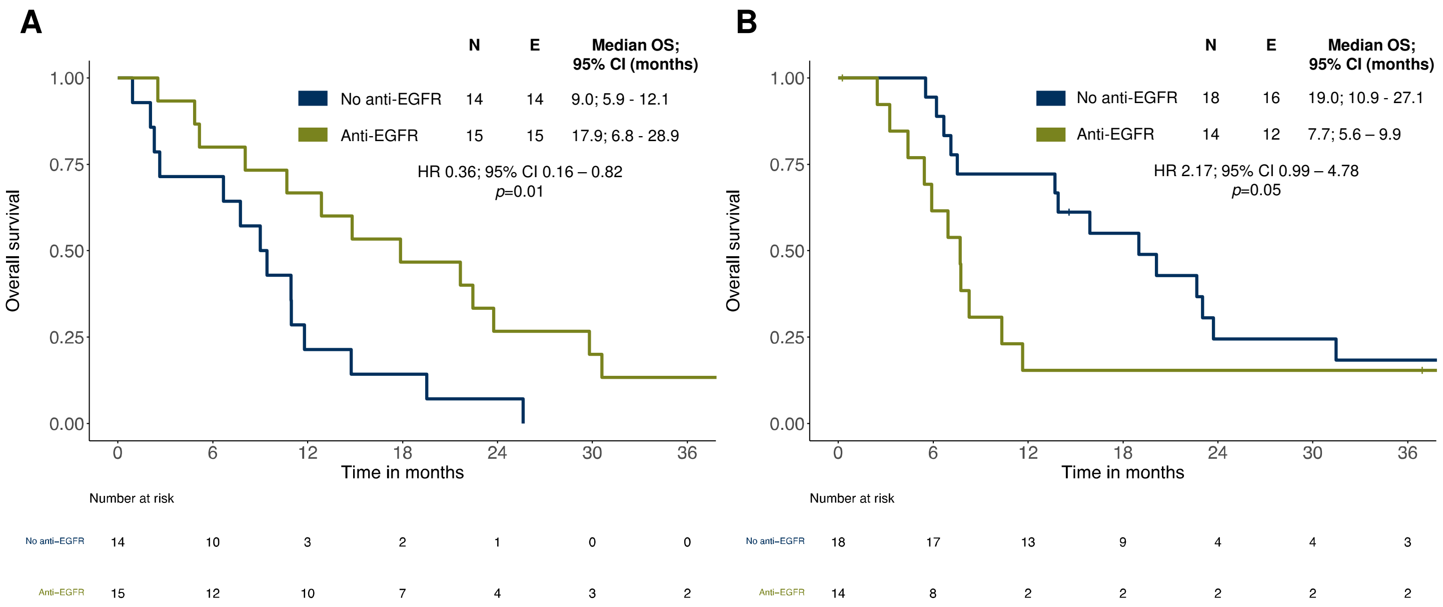

Supplementary Figure 2: OS according to treatment with or without anti-EGFR mAb regarding sidedness in the subset of patients treated in the FIRE-3 and VOLFI study

A - OS in LSPT according to treatment with or without anti-EGFR in BRAFmt mCRC

B - OS in RSPT according to treatment with or without anti-EGFR in BRAFmt mCRC
